# Using Named Entity Recognition to Identify Substances Used in the Self-medication of Opioid Withdrawal: Natural Language Processing Study of Reddit Data

**DOI:** 10.2196/33919

**Published:** 2022-03-30

**Authors:** Alexander Preiss, Peter Baumgartner, Mark J Edlund, Georgiy V Bobashev

**Affiliations:** 1 Center for Data Science RTI International Durham, NC United States; 2 ExplosionAI GmbH Berlin Germany; 3 Community Health Research Division RTI International Durham, NC United States

**Keywords:** substance abuse, opioid epidemic, opioid use disorder, self-medication, social media, Reddit, natural language processing, machine learning, network analysis, opioid, drug withdrawal, withdrawal, opioid withdrawal, mobile phone

## Abstract

**Background:**

The cessation of opioid use can cause withdrawal symptoms. People often continue opioid misuse to avoid these symptoms. Many people who use opioids self-treat withdrawal symptoms with a range of substances. Little is known about the substances that people use or their effects.

**Objective:**

The aim of this study is to validate a methodology for identifying the substances used to treat symptoms of opioid withdrawal by a community of people who use opioids on the social media site Reddit.

**Methods:**

We developed a named entity recognition model to extract substances and effects from nearly 4 million comments from the *r/opiates* and *r/OpiatesRecovery* subreddits. To identify effects that are symptoms of opioid withdrawal and substances that are potential remedies for these symptoms, we deduplicated substances and effects by using clustering and manual review, then built a network of substance and effect co-occurrence. For each of the 16 effects identified as symptoms of opioid withdrawal in the Diagnostic and Statistical Manual of Mental Disorders, Fifth Edition, we identified the 10 most strongly associated substances. We classified these pairs as follows: substance is a Food and Drug Administration–approved or commonly used treatment for the symptom, substance is not often used to treat the symptom but could be potentially useful given its pharmacological profile, substance is a home or natural remedy for the symptom, substance can cause the symptom, or other or unclear. We developed the Withdrawal Remedy Explorer application to facilitate the further exploration of the data.

**Results:**

Our named entity recognition model achieved *F*_1_ scores of 92.1 (substances) and 81.7 (effects) on hold-out data. We identified 458 unique substances and 235 unique effects. Of the 130 potential remedies strongly associated with withdrawal symptoms, 54 (41.5%) were Food and Drug Administration–approved or commonly used treatments for the symptom, 17 (13.1%) were not often used to treat the symptom but could be potentially useful given their pharmacological profile, 13 (10%) were natural or home remedies, 7 (5.4%) were causes of the symptom, and 39 (30%) were other or unclear. We identified both potentially promising remedies (eg, gabapentin for body aches) and potentially common but harmful remedies (eg, antihistamines for restless leg syndrome).

**Conclusions:**

Many of the withdrawal remedies discussed by Reddit users are either clinically proven or potentially useful. These results suggest that this methodology is a valid way to study the self-treatment behavior of a web-based community of people who use opioids. Our Withdrawal Remedy Explorer application provides a platform for using these data for pharmacovigilance, the identification of new treatments, and the better understanding of the needs of people undergoing opioid withdrawal. Furthermore, this approach could be applied to many other disease states for which people self-manage their symptoms and discuss their experiences on the web.

## Introduction

### Background

Withdrawal symptoms are major contributing factors of continued opioid misuse and relapse among those who attempt to quit [1-5]. Opioid-related withdrawal can be severe and may last for a week or longer. The symptoms often include body ache, diarrhea, nausea and vomiting, profuse sweating, insomnia, and loss of appetite [6,7]. Many patients relapse to using opioids to alleviate the symptoms. The medical treatment of withdrawal is a critical target in opioid treatment—the National Institute on Drug Abuse identified finding new treatments for opioid use disorders as its highest priority [8].

Clinicians use a variety of treatments for opioid withdrawal symptoms. Currently, opioid agonists such as methadone and buprenorphine are the most common treatments for opioid use disorder, and these are often tapered, during which time withdrawal symptoms may occur. These symptoms are often managed with standard treatments such as loperamide for diarrhea, ibuprofen for body aches, and odansetron for nausea. In 2018, the US Food and Drug Administration (FDA) approved Lucemyra (lofexidine) as the first nonopioid medication focused specifically on treating withdrawal symptoms [9,10]. At the same time, some physicians prescribe medications off-label (eg, baclofen and clonidine) to treat withdrawal. Often, these medications are used for relief of specific symptoms, such as nausea, diarrhea, or body aches. For some of these off-label treatments, the evidence base is good, but it is not as strong for others [11-15].

Although only a fraction of opioid users seek professional help to mitigate withdrawal symptoms, many seek advice from other opioid users through web-based forums and blogs [16-18]. Opioid users are actively experimenting with alternative treatments to alleviate their withdrawal symptoms. These *remedies* include use of over-the-counter medications such as loperamide for diarrhea; more experimental medication trials such as supplements (eg, vitamins and herbs); and other methods such as meditation, yoga, and acupuncture. Some of these alternative treatments are controversial (eg, the use of the opioid-containing food supplement kratom). The self-treatment practices of opioid users are poorly understood.

Social media offers unique insights into millions of web-based conversations about withdrawal remedies and can be analyzed using machine learning techniques. The broad involvement of the middle-class population in the opioid epidemic combined with the popularity of social media and the availability of smartphone devices has made web-based discussion of opioid use common. Several outlets provide searchable and analyzable information suitable for research: web-based forums such as Reddit and Bluelight, smaller personal blogs, support groups, and treatment centers, as well as Twitter. The amount of information about drug use and drug recovery contained in discussion forums is unparalleled; nowhere else is it possible to obtain such rich information about drug use and drug recovery practices. Recent studies have analyzed forum data related to opioid recovery [19], buprenorphine [20], marijuana [21], social networking [22], and emerging trends in drug use [23]. Others have shown that web-based discussion of opioids correlates with key surveillance metrics, such as synthetic opioid death rates, and could be used as a leading indicator [24]. Although studies have begun to use these sources, such information remains underused. There have been no assessments of substances used for relieving withdrawal symptoms.

### Objectives

The purpose of this study is 2-fold: (1) to validate a methodology that uses social media (Reddit posts) to investigate these self-treatment practices and (2) to better understand these practices, such as what is being used and what the consequences are of such self-help. We note that a validated methodology that uses Reddit posts to understand issues such as self-medication could have utility for a number of physical and behavioral disorders.

In our study, we focus on the following two Reddit discussion forums: *r/opiates* (“discussion of all things related to the narcotics known as opiates, from harm-reduction to pharmacology”) and *r/OpiatesRecovery* (“...a group of people dedicated to helping each other kick the habit”) [16,18]. Both forums are dedicated to open dialogue about opioid use, often with the intent of helping current and past users recover. As a part of these discussions, users often share their experiences with formal treatments and alternative treatments to mitigate the effects of withdrawal.

Our primary objective is to validate a methodology for identifying substances used to treat withdrawal symptoms from the discussions on these forums. There are no validation standards to relate discussions to the actual prevalence, incidence, and more detailed representative epidemiology of use, in large part because the forums are anonymous. At the same time, discussion forums can provide insights on the general knowledge among people who use opioids regarding the pharmacology of drugs that they are prescribed, that they buy over the counter, or that they obtain illicitly. We implicitly assume that, because of the large volume of discussions on social media, the strongest signals of remedies associated with prominent withdrawal symptoms would be clinically useful. We further expect that *common knowledge* would be more prevalent than *urban myths*. In addition to validating the methodology, we intend to demonstrate its utility for identifying clearly harmful approaches and discovering new, potentially useful remedies for withdrawal symptoms. If successful, such an approach may be useful to investigate other related questions about substance use, temporal trends, polydrug use, and other disorders where people self-manage their symptoms and discuss their experiences on the web.

Recent advances in natural language processing (NLP) have enabled researchers to identify and extract information from large amounts of text, including useful information about potential remedies. NLP has been applied to an array of problems across the health care and public health fields [25], including many aspects of the opioid epidemic [19]. Using named entity recognition (NER), we train a model on a subsample of annotated discussions from both subreddits and then apply the model to extract entities from the rest of the data. We train the NER model to identify *substances* and *effects,* where effects are *the result of using or not using a substance*. In this analysis, we focus on the effects that are symptoms of opioid withdrawal and substances that are potential remedies for those withdrawal symptoms. Thus, effects are categorized as (1) symptoms of opioid withdrawal in the Diagnostic and Statistical Manual of Mental Disorders, Fifth Edition (DSM-5), (eg, body aches); (2) effects of opioid use (eg, euphoria); (3) a medical symptom not falling into categories 1 or 2; or (4) other or unclear. Finally, we conducted a validation of the strongest signals in the data. We also developed a withdrawal remedy database and an exploration-discovery tool to assist clinicians and the pharmaceutical industry to better serve the needs of people who use opioids.

This paper is structured as follows. First, we describe the data, entity definitions, and approaches for data collection. We then describe the process for iteratively training an NER model, using it to assemble an extensive database of opioid-related substances and effects and cleaning the resulting data. Next, we describe a network analysis approach to structure the database as a network of substance and effect co-occurrence and the development of a web application tool for exploring the database. Finally, we describe the validation process, where we systematically assess some of the strongest signals in the data. We conclude with a discussion of the value of our approach, database, and tool and consider the next steps in this research.

## Methods

### Data

#### Data Acquisition and Preparation

Reddit is a public social media site comprising communities called subreddits, which organize content based on interest. A submission to Reddit is called a *post.* A post can be a link to a website outside of Reddit or a piece of text for discussion. In the latter case, this is called a *selfpost*, and the text is called *selftext.* On a post, other Reddit users can provide threaded comments. Posts and comments are archived via the pushshift service [26], and a publicly available copy of the pushshift Reddit data set is also available via the Google BigQuery platform. Using BigQuery, we downloaded all available posts and comments from the *r/opiates* (when referring to the subreddits, we adopt the nomenclature of prefixing them with *r/*) and *r/OpiatesRecovery* subreddits (the corpus), which at the time of acquisition (August 2019) extended until April 2019. In October 2020, we downloaded additional data covering May-December 2019 directly from pushshift.io using the *PushshiftRedditDistiller* package [27].

A submission on Reddit contains three possible sources of text for analysis: the title of the post, the *selftext* of the post (if it is a *selfpost*), and the threaded comments for a post. As our goal was to detect mentions of our entities within longer-form text and post titles and *selftext* often contain short phrases or incomplete sentences, we focused only on the comments that appear as discussions on a post and did not use text from post titles or *selftext* for analysis.

#### r/opiates and r/OpiatesRecovery Subreddits

We focused on content from two communities related to opioid use*:* the *r/opiates* and *r/OpiatesRecovery* subreddits. The *r/opiates* subreddit was created on June 24, 2009, and *r/OpiatesRecovery* was created on February 16, 2012. As of June 25, 2021, the *r/opiates* subreddit had 124,696 members, and *r/OpiatesRecovery* had 31,522 members [16,18].

Some basic statistics about the comments within each subreddit are presented in Table 1. Users have the ability to delete specific comments or delete their accounts and all comments, so both the count and nondeleted count are presented.

**Table 1:** Summary of comments from *r/opiates* and *r/OpiatesRecovery*.

Item	Subreddit		Total
	*r/opiates*	*r/OpiatesRecovery*	
First comment, date; time	April 8, 2010; 4:10 AM	February 16, 2012; 5:19 AM	N/A^a^
Last comment, date; time	December 31, 2019; 11:57 PM	December 31, 2019; 11:58 PM	N/A
Count	3,650,602	341,598	3,992,200
Count nondeleted	3,446,046	326,729	3,772,775

^a^N/A: not applicable.

### NER Model

We framed our NLP task as an NER problem to identify possible remedies and their effects. We aimed to identify two possible types of entities: substances and effects.

- Substance: a drug, remedy, supplement, or other consumable item used to treat an effect (eg, acetaminophen to treat body aches) or induce a desired effect (eg, heroin to induce euphoria). Although we found mentions of meditation, yoga, and other nonmedicinal remedies, in this report we only focus on consumable substances.
- Effect: a negative or positive effect mentioned as a result of consuming a substance (eg, constipation caused by opioid use or constipation relieved by polyethylene glycol use), a result of not consuming a substance (eg, diarrhea caused by opioid withdrawal), or a rationale for consuming a substance (eg, diarrhea prompting loperamide use).

We used an iterative data-labeling and model-training process to generate 6507 labeled comments that formed our training data set. For details, see Multimedia Appendix 1 [28-36]. We trained our final NER model with the default spaCy (version 2.3.0; Explosion AI) [37] settings for NER models: 10 epochs with a dropout of 0.2 [38] and compounding batch. We trained the model on 80% of the data and evaluated its performance on a randomly selected hold-out set of 20%. Precision, recall, and F1 scores were calculated for exact matches of entities.

**Figure 1:**
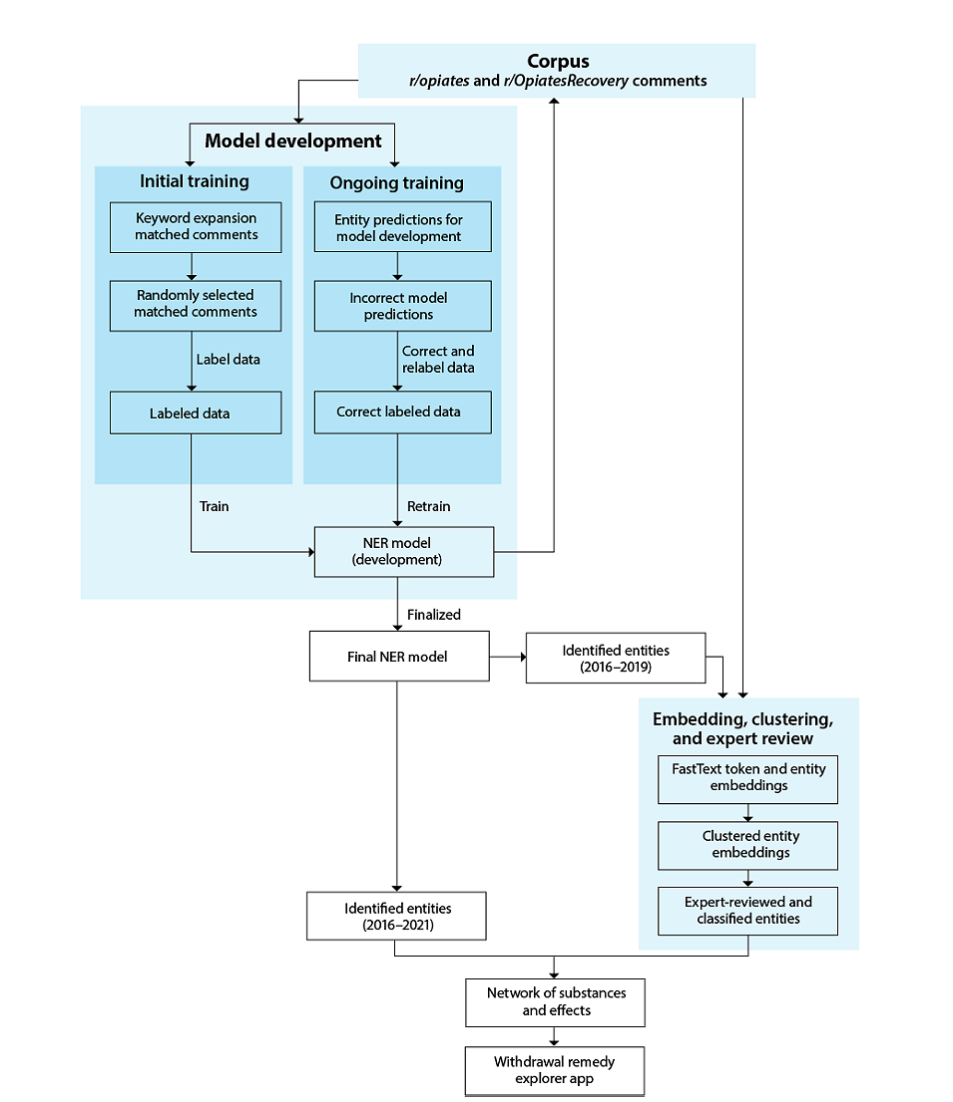
Withdrawal remedy analysis pipeline. NER: named entity recognition.

Our complete data-processing, modeling, and analysis pipeline is shown in Figure 1 and described in detail in Multimedia Appendix 1.

### Deduplication of Entities

Many of the entities identified by the model were either misspellings or semantic duplicates. Misspellings were common in the data (eg, various misspellings of a drug name). Semantic duplicates resulted when the data included semantically related words, such as synonyms, hypernyms, and hyponyms. Some of the most common semantic duplicate types in our entities included the following: (1) slang terms (eg, *fent* for *fentanyl*), (2) generic and brand name drugs (eg, *loperamide* and *Imodium*), and (3) specific and general descriptions of an effect (eg, *insomnia* and *trouble sleeping*).

To deduplicate the entities, we generated fastText word embeddings for all tokens, where tokens identified as entities were combined [39]. We then clustered entities with similar embeddings. This approach is technically related (though with opposite goals) to embedding-based methods for vocabulary expansion, which have recently been applied to opioid-related discussions from Reddit [40] (see *Detailed Methods* in Multimedia Appendix 1). After clustering, all entities in a cluster were replaced with the most common entity in the cluster. For example, *cigarettes*, *ciggarettes* [*sic*], *ciggs*, and *cigs* were clustered. The most common entity in this cluster was *cigarettes*. All occurrences of the other entities in the cluster were replaced with *cigarettes*.

### Expert Review and Classification of Entities

After clustering, we manually reviewed the deduplicated entities and conducted an additional round of deduplication and classification. Clustering was essential to reduce the number of entities and make manual review feasible. We generated separate lists of substances and the effects of these substances. The lists were reviewed by a psychiatrist (MJE) in 2 steps. First, we gave each entity a corrected name. This provided further deduplication of any slang terms or misspellings that remained after clustering. It also ensured that all entities were given a clinically accurate name. Often, these were controlled substances (eg, opioids, benzodiazepines, or prescription stimulants), illicit drugs (eg, hallucinogens or marijuana), or medications used to treat the symptoms of opioid withdrawals (eg, antiemetic or antidiarrheal medications). Second, we categorized the entities. Substances were categorized based on their pharmacological profile and use in clinical practice. Effects were categorized as (1) DSM-5 symptoms of opioid withdrawal (eg, body aches), (2) effects of opioid use (eg, euphoria), (3) a medical symptom not falling into categories 1 or 2 (eg, seizure), or (4) other or unclear (eg, sickness).

### Network Analysis

We generated a bipartite network of substance and effect co-occurrence to assess which substances were associated with these effects. We considered entities to be nodes in the network. We drew edges between nodes when a substance and effect co-occurred in a sentence. We weighted the edges by the number of times the node pair co-occurred. However, using this edge weight alone to identify significant substance-effect pairs would favor pairs where the individual probabilities of occurrence of the substance and effect were high. Therefore, we also weighted the edges using positive pointwise mutual information (PPMI) [41]. For a pair of nodes, PPMI is high when their probability of co-occurrence is high relative to their individual probabilities of occurrence.

### Application Development: Withdrawal Remedy Explorer

We built a web application called Withdrawal Remedy Explorer to provide a user-friendly way to explore the substance-effect network. Owing to its size, it was difficult to interpret the full network. Therefore, our application allows users to filter down to the ego network for a single substance or effect. Ego networks consist of a single node (ego) together with the nodes it is connected to (alters) [42] (in most cases, ego networks also include connections between the alters but, because our network is bipartite, there are no connections between entities of the same type). The application further allows users to filter according to edge count and PPMI.

### Validation Review of Pairs

We generated and validated a list of top symptom and potential remedy pairs to assess the nature of the network. For each of the 16 effects identified as DSM-5 symptoms of opioid withdrawal, we identified the top 10 nonopioid substances most strongly associated (although not necessarily causally) with the symptom. We identified these substances using a weighted average of edge count and PPMI. First, we took the natural logarithm of the edge count to reduce skewness. We then used a min–max scaler to normalize the edge count and PPMI to a range of 0 to 1. Finally, we averaged the scaled edge count and PPMI. For each withdrawal symptom, we took the 10 substances with the highest value for this calculation, omitting opioids and any pairs with an edge count <5. This produced a list of 130 strongly associated symptom and potential remedy pairs. We hypothesized that the most strongly associated pairs would be most clinically applicable. We validated each of the pairs against known medical practice and evaluated each for clinical feasibility. We then classified the pairs into categories that reflected the relationship with clinical practice, common practice, or potential harm, as follows: (1) substance is an FDA-approved or commonly used treatment for the symptom, (2) substance is not often used to treat the symptom but could be potentially useful given its pharmacological profile, (3) substance is a home or natural remedy for the symptom, (4) substance can cause the symptom, or (5) other or unclear (including cases where there was no clear connection between the potential remedy and the symptom; eg, gabapentin and fever).

## Results

### Entity Counts

Table 2 lists the top 20 most frequent substances and effects identified within the data set. As these are simply spans of text without semantic information, we can see duplicates (*fent* and *fentanyl*), slang terms (*dope* and *h*), and abbreviations (*rls*).

**Table 2:** Top 20 substances and effects extracted.

Entity			Count, n (%)^a^
**Substances (N=2,823,606)**			
	*dope*	192,385 (6.81)	
	*heroin*	160,595 (5.69)	
	*opiates*	160,244 (5.68)	
	*oxy*	95,960 (3.40)	
	*subs*	87,006 (3.08)	
	*opiate*	80,067 (2.84)	
	*methadone*	76,477 (2.71)	
	*kratom*	68,541 (2.43)	
	*fent*	68,475 (2.43)	
	*suboxone*	65,011 (2.30)	
	*h*	62,492 (2.21)	
	*sub*	57,959 (2.05)	
	*weed*	51,937 (1.84)	
	*morphine*	49,114 (1.74)	
	*benzos*	37,208 (1.32)	
	*tar*	35,573 (1.26)	
	*coke*	35,355 (1.25)	
	*xanax*	34,765 (1.23)	
	*fentanyl*	30,361 (1.08)	
	*codeine*	29,050 (1.03)	
**Effects (N=479,289)**			
	*pain*	61,783 (12.89)	
	*anxiety*	30,995 (6.47)	
	*depression*	23,579 (4.92)	
	*sleep*	22,102 (4.61)	
	*cravings*	20,319 (4.24)	
	*depressed*	11,304 (2.36)	
	*rls*	8972 (1.87)	
	*nausea*	7618 (1.59)	
	*craving*	6315 (1.32)	
	*insomnia*	5645 (1.18)	
	*mood*	5600 (1.17)	
	*puke*	5551 (1.16)	
	*seizures*	5435 (1.13)	
	*anxious*	5343 (1.11)	
	*tired*	5159 (1.08)	
	*sweating*	5099 (1.06)	
	*puking*	5029 (1.05)	
	*sickness*	4931 (1.03)	
	*sweat*	4845 (1.01)	
	*headache*	4018 (0.84)	

^a^The denominator for percentages is the total number of occurrences of all substances or effects, so percentage values do not sum to 100.

The model performs slightly better in predicting substances than effects (*F*_1_ scores of 92.059 and 81.696). Recall (92.895 for substances and 83.768 for effects) is slightly higher than precision (91.237 for substances and 79.724 for effects). Both scores are reasonable given the model architecture used and the variability and quality of the input data in comparison with common benchmark NER tasks [43].

### Deduplication and Expert Review

Both clustering and expert review greatly reduced the number of entities by removing misspellings and semantic duplicates. Clustering reduced the number of unique substances by 98.07% (from 53,730 to 1037) and reduced the number of unique effects by 95.69% (from 13,790 to 594). Relative to the number of entities remaining after clustering, expert review reduced the number of unique substances by 55.8% (from 1037 to 458) and reduced the number of unique effects by 60.4% (from 594 to 235). Our final count of unique entities was 458 substances and 235 effects.

Effects were classified into 4 categories. Their frequencies are shown in Table 3.

**Table 3:** Frequency of effects by category (N=235).^a^

Effect	Values, n (%)
DSM-5^b^ symptom of opioid withdrawal	17 (7.2)
Effect of opioid use	17 (7.2)
Not a DSM-5 symptom of opioid withdrawal or effect of opioid use	153 (65.1)
Other or unclear	48 (20.4)

^a^Percentages may not add up to 100 because of rounding.

^b^DSM-5: Diagnostic and Statistical Manual of Mental Disorders, Fifth Edition.

Substances were classified into 71 pharmacological categories. The four most common were opioid (71/458, 15.5%); other (60/458, 13.1%); vitamin, supplement, or herb (42/458, 9.2%); and food or drink (29/458, 6.3%). Multimedia Appendix 2 includes frequencies for all 71 categories.

### Network Analysis and Application

**Figure 2:**
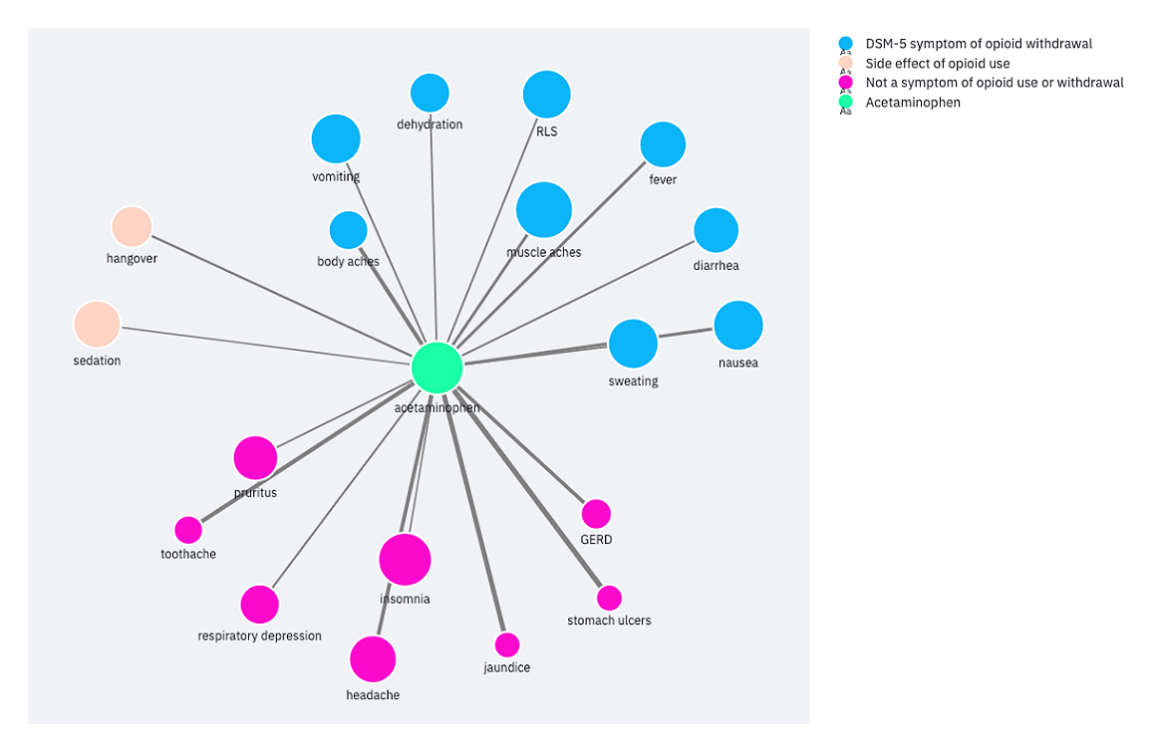
Acetaminophen ego network filtered for positive pointwise mutual information ≥1.5 and edge count ≥10. DSM-5: Diagnostic and Statistical Manual of Mental Disorders, Fifth Edition; GERD: gastroesophageal reflux disease; RLS: restless leg syndrome.

**Figure 3:**
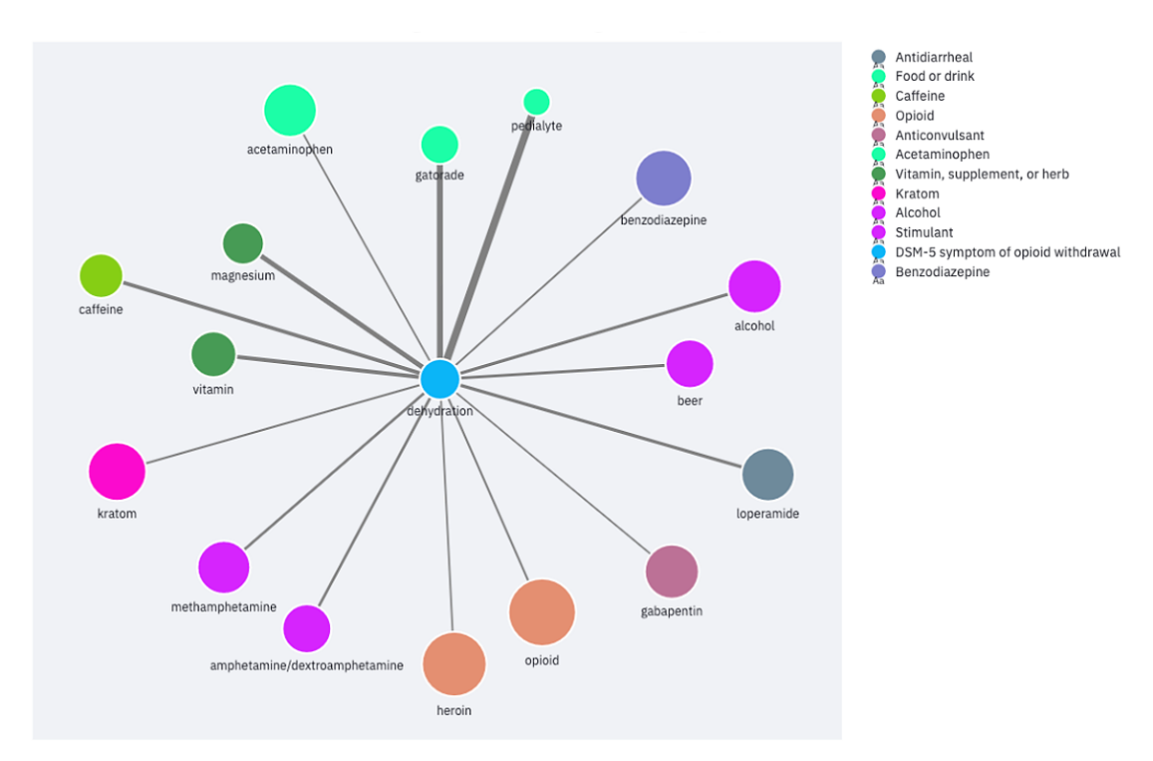
Dehydration ego network filtered for positive pointwise mutual information ≥1.5 and edge count ≥10. DSM-5: Diagnostic and Statistical Manual of Mental Disorders, Fifth Edition.

The Withdrawal Remedy Explorer application is publicly available [44]. It allows users to explore the associated substances or effects for a chosen entity. First, users can choose to view the substances or effects. Users then select an entity from a list of categorized substances or effects. This shows the ego network for the selected entity. The PPMI and edge count filters can then refine the network down to the strongest connections. For example, one could filter the network to the connections with the highest edge counts to view the most common substances associated with a symptom. Alternatively, filtering for the edges with the highest PPMI could uncover uncommon but noteworthy connections. Finally, connections with both high PPMI and high edge count are perhaps the most salient of all. Presenting the data as ego networks encourages users to identify substances or effects of interest before using the application. We considered this the most likely use case, as discussed further below. Figures 2 and 3 show example ego networks for a substance (acetaminophen) and effect (dehydration). Both ego networks are filtered for PPMI ≥1.5 and edge count ≥10.

### Validation Review of Pairs

In 64.6% (84/130) of the strongly associated symptom and potential remedy pairs, we considered the substance to be a potentially valid treatment for the symptom. This result provides evidence for the face validity of our methodology for extracting symptom–remedy pairs from Reddit. Furthermore, more strongly associated pairs were more likely to be potentially valid treatments. Of the 26 pairs in the top quintile by strength of association, 23 (88%) were potentially valid treatments for the symptom. Table 4 shows the category frequencies for the symptom and potential remedy pairs.

**Table 4:** Categorization of strongly associated symptom and potential remedy pairs (N=130).

Relationship	Substance-effect pairs, n (%)
Substance is an FDA^a^-approved or commonly used treatment for the symptom	54 (41.5)
Substance is not often used to treat the symptom but could be potentially useful given pharmacology	17 (13.1)
Substance is a home or natural remedy for the symptom	13 (10)
Substance can cause the symptom	7 (5.4)
Other or unclear (including pairs with no clear relationship)	39 (30)

^a^FDA: Food and Drug Administration.

## Discussion

### Principal Findings

In this study, we extracted information from Reddit forum posts into a database of opioid withdrawal symptoms and substances potentially used as remedies to alleviate them. Although the focus of the study was on withdrawal symptoms, we also extracted many other substances and effects associated with opioid use. We made a distinction between effects that are opioid withdrawal symptoms as defined in the DSM-5 and other effects. We validated the technical aspects of symptom and effect extraction by obtaining *F*_1_ scores that were competitive with NER benchmarks.

We used PPMI to formalize the strength of substance-effect associations. We did not use any formal statistical tests or *P* values as the data themselves contain uncertainty and the population is not clearly defined. In fact, it is likely to change over time. However, the use of PPMI allowed us to compare the strength of associations with a *by chance* association and with each other.

We used PPMI to identify a list of 130 strongly associated symptom and potential remedy pairs in which the effect was a DSM-5 opioid withdrawal symptom and the substance was a potential remedy. We validated the associations between these symptoms and potential remedies based on expert review. We discovered that roughly two-thirds (84/130, 64.6%) of the potential remedies were common treatments, potentially useful treatments, or home or natural remedies. Therefore, we observed that, for DSM-5 withdrawal symptoms, the relationship between symptom and potential remedy validates well and that *common knowledge* is more prevalent than *urban myths*. We also developed a web application that allows researchers to explore these symptoms and potential remedies through visualization and to identify associations that may be strong enough to prompt further research.

### Contributions

As demonstrated by the success of technical and expert review validations, our methodology has the potential to make significant contributions to ethnographic, clinical, and pharmacovigilance research. Specific areas include the following.

#### Understanding Potential Harm and Pharmacovigilance

Health care and public health stakeholders could benefit from knowing the types of self-medication and substitution practices that people engage in to help alleviate withdrawal symptoms, in part because those practices could lead to potential problems (eg, cardiovascular complications, medication contraindications, hospitalization, and even death). Analysis of social media discussions can rapidly inform prevention and harm-reduction activities related to new and potentially harmful beliefs and activities. This can enable stakeholders to monitor temporal trends and emerging fads. In fact, it was shown that increases in web-based posts about synthetic opioids preceded an increase in synthetic opioid death rates [45]. For example, in our study, we identified a strong association between antihistamines and restless leg syndrome (RLS). This could be a potential harm as the use of antihistamines as a remedy for RLS could in fact worsen RLS [46]. Our Withdrawal Remedy Explorer application allows one to screen for off-label use of many prescription drugs and thus assist in pharmacovigilance. Although such screening likely requires deeper follow-up with studying the actual posts, it provides a quick way to identify potential harms before they become more prevalent.

#### Identifying Home or Natural Remedies

Home or natural remedies have the potential to be inexpensive and effective measures against withdrawal symptoms. Such knowledge could help large numbers of struggling users, especially those who are not yet ready to receive treatment from a physician and who prefer the anonymity of 24/7 web-based communities to seek and share help [22]. For example, in our study, we identified a strong association between nausea and ginger, a common natural remedy [47]. Exploring associations between withdrawal symptoms and other herbs, vitamins, and supplements could help identify less well-known home or natural remedies.

#### Identifying Potentially Useful New Remedies

Successful off-label use of medications could provide leads to future clinical studies on withdrawal medications [48]. For example, in our study, we identified a strong association between gabapentin and body aches. Whether gabapentin is clinically effective for the body aches associated with opioid withdrawal is unknown; we were only able to identify 1 small study (N=32) investigating gabapentin as a treatment for opioid withdrawal [49]. Our results suggest that studies on gabapentin for opioid withdrawal may be fruitful. The identification of such remedies has not been the objective of this report, but we are partnering with clinicians and pharmacologists to identify such cases.

#### Understanding Patient Needs

Understanding patients’ needs and issues that are of importance to them is critical for the development of better prevention and treatment programs. Web-based discussion forums and social media also provide mechanisms to inform and design better treatment and harm reduction programs. For example, future research could leverage the Withdrawal Remedy Explorer application to identify withdrawal symptoms, which, at least in these forums, are of the highest concern to people using opioids. In our study, 30% (39/130) of pairs fell into the *other or unclear* category. Although some of these pairs are likely the result of limitations to our methodology, this category provides opportunities for understanding users’ beliefs and practices that go beyond *common knowledge*. Such information can lead to the discovery of new remedies or to the early identification of specific needs and potentially harmful practices.

Our approach can also be applicable to other substances of abuse, especially because currently there are no FDA-approved medications to treat cocaine, methamphetamine, and many other drug disorders. Uncovering and summarizing remedy practices for these disorders could provide at least temporary help in clinical treatment practice until treatment medications are developed and approved. Finally, by examining the symptom-related remedies, we acknowledge that some symptom–remedy pairs are quite common and are not specific to opioid withdrawal (eg, the use of melatonin for insomnia or acetaminophen for body aches). The detailed analysis of differences between remedies in opioid-specific discussions and in the general population is beyond the scope of this study.

### Limitations and Future Work

Our study has several limitations. Foremost is the lack of causality in the associations between substances and effects. Without reading the posts, it is not possible to distinguish whether the effect was caused by the substance or whether the substance was used to alleviate the effect. Furthermore, we have not yet analyzed whether the remedies were helpful. An association could take many forms (eg, “gabapentin cured my body aches,” “I tried gabapentin for body aches, but it didn’t work,” or “gabapentin gave me body aches”). However, the purpose of this study was to build and validate the foundations for such analyses. More detailed and in-depth analyses could be performed on any subset of the associations identified in the data. In future work, we plan to apply additional NLP methodologies and analysis to selected combinations of substances and effects to identify whether the association was favorable.

We only focused on associations within the same sentence. This approach misses associations when the effect and substance are mentioned in different sentences (eg, “I get terrible body aches. Aspirin does nothing for me, but gabapentin helps”). In this study, we limited the approach to sentences for clarity and simplicity. With our success using single sentences, in future work we will focus on including associations spanning multiple sentences. This task is complicated by the necessity of defining the boundary of paragraphs and detangling multiple associations between multiple substances and effects across sentences. The ambiguity of free-flowing text in posts is a common challenge in the analysis of social media.

Since the time the data were collected and the models were developed, the field of NLP has progressed significantly because of the use of transformer models such as Bidirectional Encoder Representations from Transformers [50]. For general NER tasks, this has meant an improvement from an F_1_ of 86 in 2017 to >90 in 2019. At the same time, large transformer models require significant specific computational resources [51] to train and deploy in comparison with more traditional methods and, simultaneously, focus in the field has been shifting from model-centric approaches (eg, hyperparameter tuning) to data-centric approaches (eg, higher-quality labeled data) as, in many scenarios, more benefit comes from data than architecture [52]. In summary, a limitation of our work is that we were unable to use the current state-of-the-art models, but future work will evaluate potential improvements from new model architectures and improved data quality.

In our expert review and validation processes, substances, effects, and substance-effect pairs were classified into categories according to the *face value* of the terms and without detailed assessment of the underlying post text. Different reviewers could have different opinions and interpretations, leading to variability in classifications. In future work, we plan to leverage the Withdrawal Remedy Explorer tool to seek comments and corrections from other experts.

As our study builds networks of extracted knowledge, in our future work we will consider developing a knowledge graph that would allow linking the extracted entities and their networks to external knowledge bases.

Despite these limitations, we identified and validated strong signals in the data. The discovery of valid withdrawal remedies encourages us to explore more nuanced aspects of the data. The limitations of this study point us to the next steps in our research.

### Conclusions

In this study, we validated an approach to identify opioid withdrawal remedies from the web-based forum Reddit. We developed a pipeline to extract substances and effects from raw data, identified strong associations between withdrawal symptoms and potential remedies, and validated these associations. Our results demonstrate that social media and web-based forum discussions have the potential to help us understand how people treat withdrawal symptoms. This knowledge could help identify useful new treatments and potential harms and public health concerns. We also developed the Withdrawal Remedy Explorer application to facilitate deeper analysis of these data and to seek input from other researchers, clinicians, and people with lived experience. Our approach could be generalized beyond Reddit and beyond the topic of opioid withdrawal. It could be applied to many other disease states where people self-manage their symptoms and discuss their experiences on the web.

## Appendix

Multimedia Appendix 1 Detailed methods.

Multimedia Appendix 2 Categorized substance frequencies.

## Abbreviations

DSM-5: Diagnostic and Statistical Manual of Mental Disorders, Fifth Edition
FDA: Food and Drug Administration
NER: named entity recognition
NLP: natural language processing
PPMI: positive pointwise mutual information
RLS: restless leg syndrome
