# Supplementary material for: Using Named Entity Recognition to Identify Substances Used in the Self-medication of Opioid Withdrawal: Natural Language Processing Study of Reddit Data
Source: JMIR Form Res. 2022 Mar 30;6(3):e33919. doi: 10.2196/33919 (PMC9008522; doi:10.2196/33919)
Supplement: Multimedia Appendix 1 [file formative_v6i3e33919_app1.docx]

## Multimedia Appendix 1: Detailed Methods

### Data Processing

Reddit comments can be formatted and structured using the Markdown markup language, which contains additional symbols used for structuring and formatting text (i.e., bold, italics, bulleted lists). Using Python, we converted the markdown to HTML and kept only the text elements, removing the markdown syntax and maintaining the content of the comments.

### Named Entity Recognition

#### Identifying Seed Terms for Training Data Candidates

Using the comments, we trained a series of word2vec models[28] using the genism [29] Python library. A trained word-embedding model can be used to identify words that are used in similar contexts to some input keyword. Our purpose in using it was to identify a set of keywords that would serve as seed terms for identifying relevant comments from our corpus.

The three models we trained are described in the table below:

| **Model** | **Description** |
| --- | --- |
| lowercase-sg | skip-gram, documents tokenized via spaCy [37], all lowercase |
| lowercase-sci-sg | skip-gram, documents tokenized via sci-spaCy [30], all lowercase |
| lowercase-sci-bigrams-sg | skip-gram, documents tokenized via sci-spaCy, including bigrams occurring >5 times, all lowercase |

Using these models, we iteratively developed a list of seed terms with the following process:

1. Using a set of initial terms, find the number of similar terms at various thresholds from 0.99 to 0.85 similarity.
2. Determine a similarity threshold cutoff for inclusion in the keyword expansion list.
3. Add all keywords with similarity greater than the cutoff to the keyword list.
4. Repeat from step 1, except substitute single terms with the list of similar terms.
5. Terminate process when adding terms would significantly increase the keyword list or there are no terms added above 0.85 similarity.

| 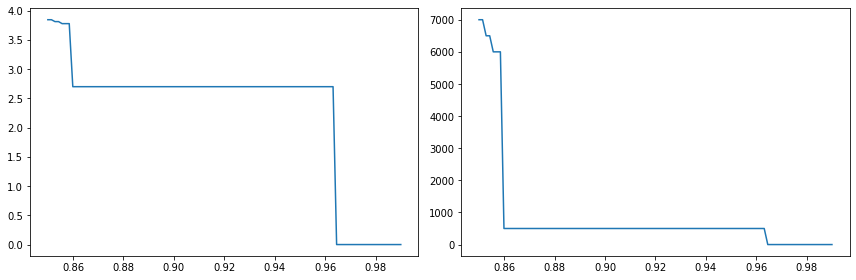 |
| --- |
| Example of threshold plots (left log10 counts, right normal counts) for the single term “wd” (abbreviation for “withdrawal”) and the number of similar terms included beyond that threshold. In this example, we would set the threshold to roughly 0.96 for inclusion in this expansion. |

Using this process we were able to generate a list of 287 seed terms for effects and 1,706 substance seed terms to assist in identifying comments that had content of interest.

#### Annotation Process

With these patterns, we used the Prodigy [31] annotation tool’s *teach* method. This method exposes an interface for quick annotation by presenting the annotator with an accept/reject decision about preselected entities generated from a combination of the patterns and an NER trained via active learning. These binary annotations were then merged into the BIO (beginning-inside-outside) format acceptable for NER. This dataset was then used with Prodigy’s correct method, which allows entity spans to be clarified by expanding or contracting the entity boundaries. The result of this process was a dataset of 3,666 entity annotations that formed an initial training dataset.

We then trained a NER model from this initial set of annotated data. After training, the model was used to predict entities on the remainder of our corpus. This dataset of predictions was then aggregated and used for error analysis to provide improvements to the model. We exported any entities that occurred over 50 times in the corpus and reviewed the entities for potential mistakes. After any entity misclassifications were identified, we generated additional training datasets by querying the corpus for comments that contained mentions of these erroneous entities. We iterated through these additional datasets using Prodigy’s *correct* method to generate additional, correct training examples that did not erroneously classify entities from the previous iteration. In addition to correcting misclassified entities, these examples often contained other entities in addition to those we were correcting for, which were also correctly labeled to provide additional data to the model.

### Deduplication of Entities

#### FastText Word Embeddings

To obtain data for clustering, we generated word embeddings for the entities using the gensim package’s implementation of Facebook’s fastText algorithm [39]. We chose fastText because of its use of subword character n-grams. Rather than assigning a distinct vector of embeddings to each word, fastText treats each word as a bag of character n-grams and assigns a vector to each n-gram. This made fastText well suited to handle both the orthographic and semantic components of this deduplication problem. Slang terms and misspellings often share character n-grams with the original term, giving them similar fastText embeddings, even when the duplicate term occurs so infrequently that distributional semantics alone may not capture its similarity to the original term.

We generated embedding vectors of length 100. We used the skip-gram algorithm with a window size of 10. The model was trained for five epochs.

#### Clustering

We then clustered the word embeddings using the HDBSCAN package’s implementation of the HDBSCAN (hierarchical density-based spatial clustering of applications with noise) algorithm [32; 33]. We chose HDBSCAN for its ability to find flexible cluster shapes and to distinguish clusters from background noise. The latter ability was particularly important since nonduplicated terms should not be clustered. We used a minimum cluster size of 3 and did not approximate the minimum spanning tree. For substances, we used cosine distance as the distance metric instead of the default Euclidean distance. We used defaults for all other parameters.

This approach created high-quality clusters but identified most entities, including many duplicates, as outliers (i.e., not in a cluster). To capture more of these duplicates, we leveraged HDBSCAN’s soft clustering feature. For each outlier, we calculated the probability of membership in each cluster. If the ratio between the highest and second-highest cluster membership probability was greater than or equal to 1.2, we assigned the outlier to the highest probability cluster. For the remaining outliers, if the entity’s frequency of occurrence was above a threshold, we formed a singular cluster from the entity. For substances, we used a frequency percentile threshold of 0.75, and for effects, 0.5. All remaining outliers were discarded.

### Application Development: Withdrawal Remedy Explorer

We used the Streamlit framework to build the Withdrawal Remedy Explorer web application, which provides a user-friendly way to explore the data as a network of connections between substances and effects.[34] The application consists of three components: underlying data sources, visualization functions, and a script to create the application. The underlying data sources are tables of nodes and edges that make up the bipartite substance-effect network discussed in the network analysis section. They include columns for the entity names, categories, edge weights, and PPMI, which enable the user to filter to an ego network. The visualization functions use the Python package Plotly to draw the ego network that the user has selected.[35] Finally, a single script controls the application. The script declares widgets to take user input, filters the data accordingly, and plots the data. We deployed the application on Streamlit Sharing, which avoids the need to provision a server and host the application. Withdrawal Remedy Explorer is open source and available on GitHub.[36]
